# Maternal mortality due to hypertensive disorders in pregnancy, childbirth, and the puerperium between 2012 and 2015 in Turkey: A nation-based study

**DOI:** 10.4274/jtgga.2016.0244

**Published:** 2017-03-01

**Authors:** Bekir Keskinkılıç, Yaprak Engin-Üstün, Sema Sanisoğlu, Dilek Şahin Uygur, Hüseyin Levent Keskin, Selma Karaahmetoğlu, Ayşe Özcan, Meral Esen, Afra Alkan, Aysun Kabasakal, İrfan Şencan

**Affiliations:** 1 Turkish Public Health Agency, Preliminary Investigation Committee for Maternal Deaths, Ministry of Health, Ankara, Turkey

**Keywords:** Hypertensive disorders, maternal mortality, Turkey

## Abstract

**Objective:**

To analyze maternal deaths in Turkey due to hypertensive disorders.

**Material and Methods:**

In this retrospective study 812 maternal deaths were analyzed. Maternal demographic features, presence of antenatal care, medical and obstetric history, mode of delivery, and use emergency antihypertensive therapy were recorded. The delay model for each case was investigated.

**Results:**

Hypertensive disorders accounted for 15.5% (n=126) out of all maternal mortality. They were the third most frequent cause among all causes and the 2^nd^ among direct causes of maternal deaths. Sixty-one (48.4%) cases were in severe preeclampsia or pre-existing hypertensive disorder with increased/superimposed proteinuria, 30.1% were in eclampsia, 9.5% cases were diagnosed as hemolysis, elevated liver enzymes, low platelet count syndrome, and 11.1% in pre-existing hypertension complicating pregnancy, childbirth, and puerperium without increased or superimposed proteinuria. The median age was 32 years, 37.3% women were ≥35 years. All deaths except for 2 cases occurred during the postpartum period. Twenty-three percent of deaths occurred in the first 48 hours postpartum, and 51.6% between 8-42 days. Intracranial hemorrhage was the major final cause of death with a rate of 41.3%. With the exception of fifteen patients with intracranial hemorrhage, emergency antihypertensive agents were not implemented in optimal dose and/or duration. A first and/or third delay was identified in 36.5% of cases.

**Conclusion:**

Approximately one third of maternal death due to hypertensive disorders could be prevented. The importance of acute antihypertensive treatment should be emphasized because of most frequent cause of death was intracranial hemorrhage.

## INTRODUCTION

One of the three health-related goals of the Millennium Developmental Goals was the reduction of maternal mortality by three-quarters by the year 2015 (1). Maternal deaths are not uniformly distributed throughout the world and most maternal deaths occur in undeveloped regions such as sub-Saharan Africa (1). High maternal mortality ratios (MMR) are related with development and cultural factors that are not easy to change. The MMR of Turkey between 2007 and 2009 was 19.7 per 100,000 live births (2). Most maternal deaths are preventable and a review of the underlying clinical and social risk factors is important to decrease the number of these deaths (3). It has been estimated that 74% of maternal mortality can be averted if all women receive appropriate emergency obstetric care (4).

Hypertensive disorders in pregnancy, childbirth, and the puerperium are one of the most frequent causes of maternal and perinatal mortality (5). There is no proven effective method for the prevention of preeclampsia.

Our aim was to analyze maternal deaths in Turkey due to hypertensive disorders in pregnancy, childbirth, and the puerperium.

## MATERIAL AND METHODS

In this retrospective study, medical records of all pregnancy-associated deaths recorded in Turkey between 2012 and 2015 were reviewed. The Turkish Statistical Institute (TURKSTAT) has been collecting data on the number of deaths and causes of death using the vital registration (VR) system since 2009 in details of ICD-10 codes, and underlying cause is the main concern as the World Health Organization (WHO) suggests. The VR system of TURKSTAT collects data through forms that include a check box to mark whether the death was a maternal death. Maternal deaths are also debated by the Ministry of Health (MoH) because the MoH assigns a committee of doctors and specialists to discuss suspicious maternal deaths in detail to determine whether the death fulfilled the WHO maternal death criteria. The MoH made an act that stated that death notifications would no longer be received from the field through paper forms but through the death notification system so that deaths were registered to the VR. The VR system works well in terms of completeness and accuracy of data.

The underlying cause as “disease or injury that initiated a chain of events, which lead to death” was noted. Deaths related to hypertensive disorders of pregnancy were evaluated. All maternal deaths in Turkey are reported to the Preliminary Investigation Committee for Maternal Deaths at the MoH of Turkey. A team of clinicians including 2 nurses/midwives, 1 perinatologist, 2 obstetricians, 1 internal medicine specialist, and 1 anesthetist evaluate the medical records in a group setting in order to arrive at consensus on clinical determinations. Identifying the cause and preventability of maternal mortality includes medical hospital records, death certificates, autopsy reports, local and national registries, and verbal autopsy. Each case is analyzed separately in the first month after death occurs.

The WHO application of “ICD-10 to deaths during pregnancy, childbirth, and the puerperium: ICD-MM” manual was used for the definitions (6). Under the ICD-MM, maternal death is defined as the death of a woman while pregnant or within 42 days of termination of the pregnancy, irrespective of the duration and site of the pregnancy, and of any cause related to or aggravated by the pregnancy or its management with the exception of accidental or incidental causes. Late maternal deaths (more than 42 days but less than 1 year after the termination of pregnancy) were excluded in the present study. MMR was calculated as the number of maternal deaths during the given time period per 100,000 live births during the same time period.

A death was classified as preventable by consensus of the expert committee. An event was considered preventable if one of the three delays was reported (7, 8). Phase 1 delay: delay in deciding to seek appropriate medical help for an obstetric emergency, phase 2 delay: delay in reaching an appropriate health facility; phase 3 delay: delay in receiving adequate emergency obstetric care when a facility was reached.

In the present study hypertensive disorders in pregnancy, childbirth, and the puerperium that caused maternal death were distributed into four groups; 1- Pre-existing hypertensive disorder complicating pregnancy, childbirth, and the puerperium with increased/superimposed proteinuria and severe preeclampsia; 2- Hemolysis, elevated liver enzymes, low platelet count (HELLP) syndrome; 3- Eclampsia; and 4- Pre-existing hypertension complicating pregnancy, childbirth, and the puerperium without increased/superimposed proteinuria.

Data on maternal demographic features, presence of antenatal care, medical and obstetric history, mode of delivery, and medication (including emergency antihypertensive therapy and eclampsia prophylaxis with magnesium sulphate) were recorded. This investigation was reviewed and approved by the Public Health Agency of the MoH of Turkey.

Data were entered into a database and analyzed. The Statistical Package for the Social Sciences statistical software package version 16.0 (SPSS Inc., Chicago, IL, USA) was used for the analyses. The results were presented as frequencies, percentages, and descriptive summary statistics. The Chi-square test was used for comparison of the categorical data between the groups. P<0.05 was considered statistically significant.

## RESULTS

In Turkey, a total of 812 maternal deaths were recorded between 2012 and 2015. In this period, hypertensive disorders in pregnancy, childbirth, and the puerperium accounted for 15.5% (n=126) of all maternal mortality. They were the third most frequent cause among all causes after diseases of the circulatory system complicating pregnancy, childbirth, and the puerperium (n=180, 22.2%) and obstetric hemorrhage groups (n=146, 18.0%), and they were second among direct causes of maternal death (Table 1).

The distribution of hypertensive disorders in pregnancy, childbirth, and the puerperium was 61 cases in severe preeclampsia or pre-existing hypertensive disorder with increased/superimposed proteinuria (48.4%), 39 in eclampsia (31.0%), 12 in HELLP syndrome (9.5%), and 14 cases in pre-existing hypertension complicating pregnancy, childbirth, and the puerperium without increased or superimposed proteinuria. The rate of severe preeclampsia or pre-existing hypertensive disorder with increased/superimposed proteinuria decreased gradually and was statistically significant from 2012 to 2015 (χ2=23.135, p=0.006) (Table 2).

Sociodemographic and clinical characteristics of the women with hypertensive disorders of pregnancy are depicted in Table 3. The ages of the 126 women with hypertensive disorders of pregnancy ranged from 19 to 48 years with a median of 32 years (mean; 31.6±6.3 years). Out of 126 women, 47 (37.3%) women were aged ≥35 years. The majority of women (n=102, 81%) were found to receive adequate antenatal care from any health care practitioners according to the WHO criteria. All women who did not receive antenatal care were illiterate (Table 3).

With the exception of 2 cases, all deaths occurred during the postpartum period. Twenty-three percent (n=29) of deaths occurred in the first 48 hours postpartum, 23.8% (n=30) occurred between 2-7 days, and 51.6% (n=65) between 8-42 days. The time interval from admission to any medical facility to death was 7.29±0.83 days. A total of 89.7% (n=113) women underwent cesarean section. The major cesarean delivery indications in the study were preeclampsia complications, i.e. hypertensive emergencies or HELLP syndrome (n=82), fetal distress (n=13), and abruption placenta (n=15). Cesarean section performed because of previous cesarean and breech presentation without any evidence of hypertension at delivery was performed in only 3 women. We report no cases of prolonged or obstructed labor.

Out of 39 maternal deaths due to eclampsia, 27 (69.2%) were diagnosed during the antenatal period, whereas 10 (25.6%) had a postnatal onset. Only 2 patients had a seizure during labor. Out of the 27 cases diagnosed during the antenatal period, 5 were diagnosed before 32 weeks of gestation, and 22 at or after 32 weeks of gestation. All women with postnatal eclampsia delivered after 32 weeks of gestation. The rate of specific use of magnesium sulfate in eclampsia was (37/39) 94.9%.

Intracranial hemorrhage was the major final cause of death with a rate of 41.3% (n=52) and was noted in 24 patients with eclampsia, five patients with HELLP syndrome, and 23 patients with severe preeclampsia or pre-existing hypertensive disorder with/without increased/superimposed proteinuria. Except for fifteen patients with intracranial hemorrhage, emergency antihypertensive agents (nifedipine, hydralasine or labetalol) were not implemented in optimal dose and/or duration before the event had occurred. Pulmonary edema was the final cause of death in 11 patients with severe preeclampsia or pre-existing hypertensive disorder with/without increased/superimposed proteinuria, and in two patients with HELLP syndrome.

When the delay models were analyzed, no delay was found during the mortality process in 80 (63.5%) cases. A phase 1 delay was determined delay in 29 (23.0%) cases, i.e. delay in seeking care by the patient, and a phase 3 delay in 19 (15.1%), i.e. preventable factors, before death occurred. In two cases, both phase 1 and 3 delays were present.

Twenty-three patients with delay in seeking care (23/29, 79.3%) were illiterate or had primary level (≤4 years) education. However, this rate was 62.5% (50/80) in cases in which no delay was identified. There was no correlation between educational status of women and phase 1 delay (p=0.099).

## DISCUSSION

Globally, an estimated 287,000 maternal deaths occurred in 2010. MMR is one of the most important public health indicators that reveals the development of a country's economy, culture, and healthcare system. The global MMR in 2010 was reported as 210 maternal deaths per 100,000 live births. Although one of the aims of MDG was to reduce the MMR by three quarters till the year 2010 with respect to the rates of 1990 the rate of reduction is still well short of the 5.5 per cent annual decline needed to meet the target (9). However Turkey has experienced an annual decline above this rate with 5.8% (9). Preeclampsia, which affects 2% of pregnancies, leads to considerable maternal and fetal morbidity and mortality (5). In a cross-sectional study designed by the WHO (10), which was conducted at health facilities in 29 countries from Africa, Asia, Latin America, and the Middle East, incidences of pre-eclampsia, eclampsia, and chronic hypertension were reported as 2.16%, 0.28%, and 0.29%, respectively. The present report provides a national estimate of the incidence of maternal mortality from hypertensive disorders in pregnancy, childbirth, and the puerperium in Turkey between 2012 and 2015. Maternal mortality due to hypertensive disorders in pregnancy, childbirth, and the puerperium was 16.4% between 2007 and 2009 in Turkey (2). Similar to the 2007- 2009 period, 15.5% of maternal deaths were due to the same reason between 2012 and 2015.

In the present study, the highest ratio of maternal deaths caused by hypertensive disorders (62.7%) was in mothers aged <35 years. All deaths except 2 occurred during the postpartum period.

Eclampsia represented approximately 31.0% of hypertensive disorders. We found high rates of administration of magnesium sulfate in eclampsia. The cesarean delivery rate was high in the study but we found that it was widely used as a life-saving procedure. In the present study, 36.5% of hypertensive deaths were classified as preventable and the key preventable factor was delay in seeking care by the patient (23%). Moodley (11) reported 507 deaths associated with hypertensive disorders in pregnancy, childbirth, and the puerperium, and similar to our results, the author showed that the most common preventable factors were patient-oriented problems, i.e. women who either presented late for antenatal care or late to hospital when they became symptomatic.

Adu-Bonsaffoh et al. (12) reported that eclampsia, acute renal failure, intracranial hemorrhage and pulmonary edema were the major immediate causes of hypertension-related maternal death in their population. Bentata et al. (13) reported hypertensive disorders were the main reason for admission to the intensive care unit. In the present study, we found that the major final cause of death in hypertensive disorders of pregnancy was intracranial hemorrhage. Intracranial hemorrhage is an overwhelming cause of death in women with hypertensive disorders, which reflects a failure of effective treatment of systolic hypertension, although the explosive nature of fulminating preeclampsia may also cause intracranial hemorrhage. Maybe these patients would have a better prognosis if a standardized clinical protocol was adopted for the management of hypertensive disorders in pregnancy. We recommend that all maternity units have clear guidelines for the management of severe preeclampsia and treatment protocols should include emergency antihypertensive agents (nifedipine, hydralazine or labetalol), which were preferred as first-line therapy for emergency therapy of acute-onset severe hypertension by the American College of Obstetricians and Gynecologists in 2015 (14). Clark et al. (15) also stated that disease-specific protocols were beneficial in the reduction of maternal mortality because of hypertensive disorders of pregnancy. They reported that a policy of automatic and emergency antihypertensive therapy for hypertensive disorders of pregnancy eliminated deaths from in-hospital intracranial hemorrhage and decreased maternal deaths. Besides using antihypertensive agents, especially fluid restriction or at least giving under the controlled limits should be addressed as specific aspects of preeclampsia management because of the risk of acute renal failure development (16).

In conclusion, more efforts are needed to decrease maternal mortality in Turkey. At present, new strategies are being developed by the MoH for reducing maternal deaths. Eradicating preventable hypertensive maternal deaths will require an improvement in educational status of women, implementation of clear guidelines for the management of acute-onset severe hypertension, and the ability to easily and immediately obtain emergency antihypertensive agents, because the most frequent cause of deaths is intracranial hemorrhage.

## Figures and Tables

**Table 1:**
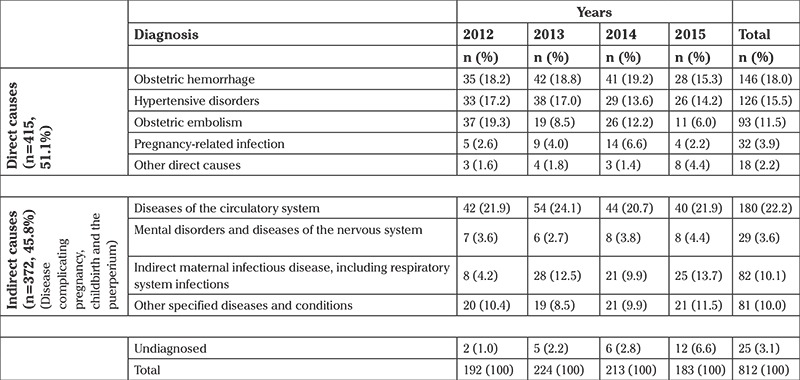
Maternal deaths based on diagnosis

**Table 2:**
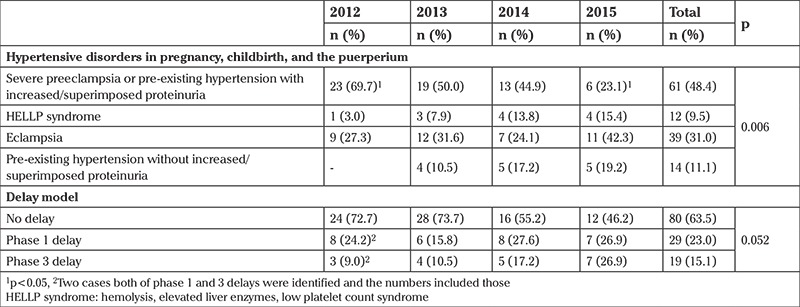
Distribution of the cases and delay models according to years

**Table 3:**
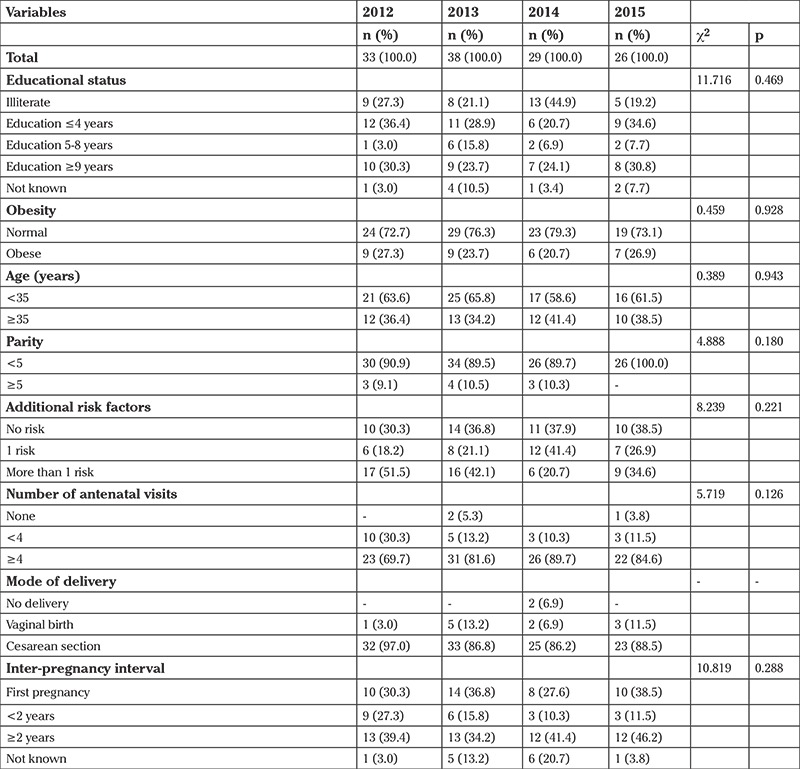
Demographic characteristics of the maternal deaths based on years
